# Non-antibiotic pharmaceuticals promote conjugative plasmid transfer at a community-wide level

**DOI:** 10.1186/s40168-022-01314-y

**Published:** 2022-08-12

**Authors:** Yue Wang, Zhigang Yu, Pengbo Ding, Ji Lu, Uli Klümper, Aimee K. Murray, William H. Gaze, Jianhua Guo

**Affiliations:** 1 Australian Centre for Water and Environmental Biotechnology (ACWEB, Formerly AWMC), The University of Queensland, Brisbane, QLD 4072 Australia; 2 Institute for Hydrobiology, Technische Universität Dresden, 01217 Dresden, Germany; 3 European Centre for Environment and Human Health, University of Exeter Medical School, Environment & Sustainability Institute, Penryn Campus, Penryn, TR10 9FE UK

**Keywords:** Antibiotic resistance, Non-antibiotic pharmaceuticals, Broad-host-range conjugative plasmid, Activated sludge, Microbial community, Reactive oxygen species

## Abstract

**Background:**

Horizontal gene transfer (HGT) plays a critical role in the spread of antibiotic resistance and the evolutionary shaping of bacterial communities. Conjugation is the most well characterized pathway for the spread of antibiotic resistance, compared to transformation and transduction. While antibiotics have been found to induce HGT, it remains unknown whether non-antibiotic pharmaceuticals can facilitate conjugation at a microbial community-wide level.

**Results:**

In this study, we demonstrate that several commonly consumed non-antibiotic pharmaceuticals (including carbamazepine, ibuprofen, naproxen and propranolol), at environmentally relevant concentrations (0.5 mg/L), can promote the conjugative transfer of IncP1-α plasmid-borne antibiotic resistance across entire microbial communities. The over-generation of reactive oxygen species in response to these non-antibiotic pharmaceuticals may contribute to the enhanced conjugation ratios. Cell sorting and 16S rRNA gene amplicon sequencing analyses indicated that non-antibiotic pharmaceuticals modulate transconjugant microbial communities at both phylum and genus levels. Moreover, microbial uptake ability of the IncP1-α plasmid was also upregulated under non-antibiotic pharmaceutical exposure. Several opportunistic pathogens, such as *Acinetobacter* and *Legionella*, were more likely to acquire the plasmid conferring multidrug resistance.

**Conclusions:**

Considering the high possibility of co-occurrence of pathogenic bacteria, conjugative IncP1-α plasmids and non-antibiotic pharmaceuticals in various environments (e.g., activated sludge systems), our findings illustrate the potential risk associated with increased dissemination of antibiotic resistance promoted by non-antibiotic pharmaceuticals in complex environmental settings.

Video abstract

**Supplementary Information:**

The online version contains supplementary material available at 10.1186/s40168-022-01314-y.

## Background

The rapid dissemination of antibiotic resistance among bacterial communities is mainly driven by horizontal gene transfer (HGT) [1, 2]. In particular, broad-host-range, conjugative plasmids that carry antibiotic resistance genes (ARGs) are critical for the spread of antibiotic resistance, as they are able to transfer into and be maintained in distantly related hosts, hence transferring genes across bacterial phyla and even domains of life [3–5]. Plasmid-mediated conjugation happens in natural environments spontaneously and at slow rates [6]. However, the intensive use of antibiotics likely stimulates conjugative transfer of and selection for antibiotic resistant plasmids in both clinical and environmental settings [7–9], thus inducing evolutionary community changes [10].

In natural environments, not only antibiotics, but also other xenobiotic contaminants are present at elevated levels relative to pristine environments. For example, non-antibiotic pharmaceuticals, which occupy 95% of overall drug consumption [11], enter the environment via human excretion, domestic sewage, husbandry manure, hospital, and manufacturing wastewaters [12, 13]. Recently, it was reported that more than 200 non-antibiotic pharmaceuticals could impose antibiotic-like effects on selected human gut bacterial strains [14]. Further, we previously showed that several commonly consumed non-antibiotic pharmaceuticals (e.g., nonsteroidal anti-inflammatory drugs) could promote the spread of antibiotic resistance in single bacterial strains [15, 16]. However, when considering the wide variety of bacteria co-existing in natural ecosystems as members of complex microbial communities [17], it remains unknown whether these non-antibiotic pharmaceuticals can also promote the dissemination of antibiotic resistance at a community-wide level.

**Fig. 1:**
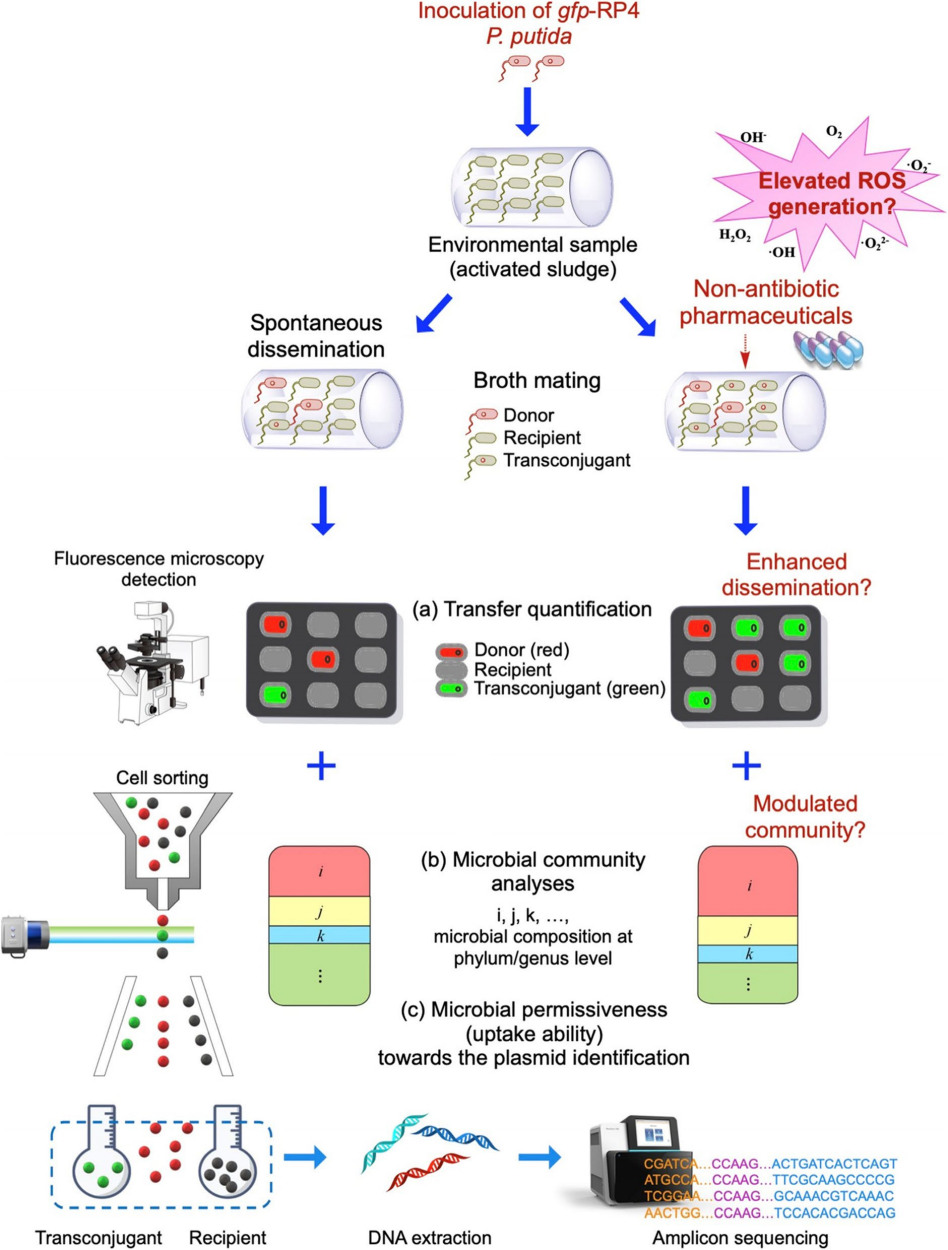
Experimental design and methods used in this study. Fluorescently *mCherry*-tagged environmental bacterium *Pseudomonas putida* hosting the broad-host-range self-transmissible IncP-*α* plasmid RP4 was added to the activated sludge community in liquid phase mating assays. The RP4 plasmid hosts multiple resistance genes against ampicillin, kanamycin, and tetracycline, and was here used in a *gfp*-tagged form. The expression of GFP was chromosomally repressed in the donor strain, and only expressed upon success transfer in the recipient strain. The broth mating was exposed to different non-antibiotic pharmaceuticals. **a** The transfer was visualized by a fluorescence microscopy, followed by quantification and calculation of transfer ratio. **b** Fluorescence-activated cell sorting (FACS) of putative recipients and transconjugants and subsequent 16S rRNA gene amplicon sequencing were employed to investigate shifts in recipient and transconjugant communities upon exposure to these non-antibiotic pharmaceuticals. **c** Microbial permissiveness (uptake ability) towards the plasmid were also identified

To this end, we explored the potential of commonly consumed non-antibiotic pharmaceuticals to alter plasmid-mediated antibiotic resistance HGT dynamics in a complex, activated sludge microbial community. Activated sludge is a suitable model environment, as it contains a high diversity of human-associated as well as environmental bacteria, and simultaneously enriches environmental pollutants [18, 19]. To track and quantify conjugative transfer, the fluorescently *mCherry*-tagged environmental bacterium *Pseudomonas putida* hosting the broad-host-range self-transmissible IncP-*α* plasmid RP4 [20], was added to the activated sludge community in liquid phase mating assays. The RP4 plasmid hosts multiple resistance genes against ampicillin, kanamycin, and tetracycline, and was here used in a *gfp*-tagged form [20]. The expression of GFP was chromosomally repressed in the donor strain, and only expressed upon transfer in the recipient strain, hence allowing visualization, quantification, and isolation of green fluorescent transconjugants [21]. In addition, we performed mating experiments across gradients of six commonly consumed non-antibiotic pharmaceuticals, including an anticonvulsant (carbamazepine), two nonsteroidal anti-inflammatory drugs (ibuprofen and naproxen), a lipid-lowering drug (gemfibrozil), a *β*-blocker (propranolol), and an X-ray contrast agent (iopromide) to test if they could promote plasmid transfer within the complex community. These non-antibiotic pharmaceuticals are frequently detected as pollutants in various environments such as wastewater, groundwater, surface and drinking water, with concentrations ranging from nanograms to milligrams per liter [22, 23]. After exposure to these non-antibiotic pharmaceuticals, conjugative transfer was visualized and quantified by fluorescence microscopy. Bacterial reactive oxygen species (ROS) generation, a common result of exposure to these non-antibiotic pharmaceuticals [16] was measured by flow cytometry. Furthermore, fluorescence-activated cell sorting (FACS) of putative recipients and transconjugants and subsequent 16S rRNA gene amplicon sequencing were employed to investigate shifts in recipient and transconjugant communities upon exposure to these non-antibiotic pharmaceuticals. This allowed identification of genera most likely to take up the broad-host-range plasmid and thus become resistant to multiple antibiotics, as well as that fraction with increased permissiveness (uptake ability) towards the plasmid when exposed to non-antibiotic pharmaceuticals (Fig. 1). These findings offer insights into the dissemination of antibiotic resistance promoted by non-antibiotic pharmaceuticals at a community-wide level.

## Methods

### Donor strain and culture condition

*Pseudomonas putida* KT2440 carrying conjugative RP4 plasmid was used as the donor bacterial strain. The RP4 plasmid is a 60 Kb, broad-host-range, low-copy-number, conjugative IncP1-α plasmid, harboring resistance against the antibiotics ampicillin, kanamycin, and tetracycline [24]. Both the donor strain and the RP4 plasmid were constructed previously [20, 25]. The RP4 plasmid was tagged with a *gfpmut3b* gene under the control of a LacI^q^-repressible P_A1/O4/O3_ promoter [25]. The donor strain *P. putida* KT2440 was chromosomally tagged with a constitutively expressed *lacI*^*q*^ and *mCherry* genes using the mini-Tn7 tagging system [20]. Therefore, plasmid-encoded *gfp* expression is repressed in the donor strain, but can be expressed upon successful transfer to a recipient bacterial strain, which lacks the chromosomal *gfp*-repressor. The donor strain was cultured in lysogeny broth (LB) broth (pH 7.0) supplemented with 10 mg/L tetracycline to select for plasmid carriage at 30 °C overnight under shaking conditions. After culturing, the donor strain was harvested by centrifugation and washed twice with phosphate-buffered saline (PBS, pH = 7.2). Meanwhile, the stability of RP4 plasmid in the donor cell was examined (Text S1) [26] and we found that RP4 plasmid was stably maintained during 24-h mating (Figure S1).

### Recipient microbial community

The recipient activated sludge microbial community was harvested from a lab-scale activated sludge bioreactor (Australian Centre for Water and Environmental Biotechnology, The University of Queensland, Australia). The bioreactor is a sequencing batch reactor (SBR) fed with raw wastewater, where the total chemical oxygen demand (TCOD) was 335 ± 63 mg/L and 102 ± 33 mg/L in the influent and effluent, respectively, and ammonium was 44.7 ± 4.5 mg N/L and 0.5 ± 0.2 mg N/L in the influent and effluent, respectively. A total of 100 mL of activated sludge were sampled and treated by sonication at 80 J for 30 min with alternating pulses and pauses of equal duration every second (Branson Sonifier 450), to achieve a homogeneous phase that allows extraction of the bacterial fraction. Notably, the sonication process did not affect activated sludge bacterial performance, as the oxygen uptake rate [27] did not change significantly before and after sonication (before 12.0 ± 0.4 mg O_2_/L/h, after: 12.5 ± 0.6 mg O_2_/L/h, *P* = 0.30). After 10 min settling of the homogenized activated sludge sample, 50 mL of supernatant was collected to serve as the recipient community (total suspended solids, TSS, 710 ± 10 mg/L).

### Broth mating assay

The donor strain was re-suspended in PBS and diluted to an initial concentration of 10^7^ CFU/mL based on OD_600_ values (the relationship between OD_600_ and bacterial concentration was predetermined). To simulate real environments, and since some bacteria present in activated sludge are not culturable by standard methods [28], the broth mating assay was carried out in medium identical to the feed of the bioreactor from which the activated sludge sample was taken. The conjugative broth mating system was established by mixing the donor and the collected recipient community with a volume ratio of 1:1.

During the mating, donor strain *P. putida* [29] with chromosomal *mCherry* appeared red, while recipient activated sludge bacteria appeared green after successful uptake and expression of GFP encoded by the *gfp*-tagged conjugative plasmid. Notably, activated sludge may display autofluorescence due to the major sludge floc component-extracellular polymeric substances [30], but the sonication process can disrupt these flocs and eliminate autofluorescence [31]. Thus, the recipient bacteria used in this study appeared colorless initially.

The selected commonly detected non-antibiotic pharmaceuticals (carbamazepine, gemfibrozil, ibuprofen, iopromide, naproxen, and propranolol) were dissolved in the solvent Dimethyl sulfoxide (DMSO) and dosed into the mating system, with final concentrations of 0.5 (environmentally relevant [22, 32, 33]), 5, or 50 mg/L. Although 5 and 50 mg/L are not environmentally relevant concentrations, the reason to include them was to investigate if any dosage-dependent patterns exist under exposure to these pharmaceuticals. As a non-pharmaceutical control, the pure solvent DMSO was added at equal volumes as used in the pharmaceutical positive mating assays. After 24 h mating at room temperature (25 ± 5 °C) without shaking, the mating mixtures were homogenized by vortexing for 30 s and then subjected to bacterial, as well as transfer quantification and cell sorting. All experiments were conducted in at least six biological replicates.

### Quantification of conjugative transfer

Conjugation events were visualized by a confocal laser scanning microscope (CLSM, LSM710, Zeiss, Germany) and Zen Software (Zeiss, Germany). In detail, 96-well plates (Costar®, USA) containing 40 μL of the mating mixtures were imaged on the reverse microscope. Notably, 40 μL was the volume that could cover the bottom of each well as the single layer. The number of red (donor), green (transconjugant), and non-fluorescence (recipient) was quantified by ImageJ software (NIH). Donor and transconjugant fluorescence detections were based on excitation at 480/20 nm with emission at 525/40 nm (*gfpmut3*) and excitation at 580/25 with emission at 650/60 nm (*mCherry*), respectively. The captured images were processed by increasing contrast, subtracting background, eliminating poorly illuminated corners, and the bacterial objects were counted (Zen Software, Zeiss, and ImageJ Software, NIH). The conjugative transfer ratio was finally calculated as the quantified number of transconjugants divided by the number of total recipient bacteria. Simultaneously, the treatment with antibiotic chloramphenicol was also conducted as a positive control (Text S2; Figure S2).

### Detection of bacterial ROS production

ROS production of the donor as well as the recipient community was measured as a function of exposure to non-antibiotic pharmaceuticals. A CytoFLEX S flow cytometer (Beckman Coulter, USA) and the 2′,7′-dichlorofluorescein diacetate (DCFDA, abcam®, UK) dye were applied to detect ROS production according to previous studies [34, 35]. The *P. putida* KT2440 doner with the RP4 plasmid was cultured overnight, washed twice with PBS, and resuspended in PBS to reach a concentration of 10^6^ CFU/mL. The recipient microbial community was retrieved from activated sludge as previously described, filtered and diluted 10- fold in PBS. The donor and recipient microbial community were then stained with DCFDA (final concentration 20 μM) for 30 min in the dark separately, and exposed to different concentrations of non-antibiotic pharmaceuticals. 1.5% H_2_O_2_ was set as positive control, and solvent DMSO was set as negative control. The ROS production was measured at 488 nm and data was processed in CytExpert software (Beckman Coulter, USA). Relative fold changes of ROS production were calculated as pharmaceutical-treated samples divided by the DMSO solvent control sample. The ROS detection was conducted in six biological replicates.

### Bacterial cell sorting and 16S rRNA gene amplicon sequencing

Each homogenized mating assay of donor and recipient cells was filtered through a 35-μm nylon mesh cell strainer (Falcon® round-bottom tubes with cell strainer cap), to remove large flocs. The mixtures were then diluted tenfold in PBS, followed by cell sorting on FACS (BD FACSAria III, USA). Transconjugant and recipient communities were sorted based on bacterial size, green fluorescence (for transconjugants), and the exclusion of red fluorescent donor cells according to previous protocols [20, 21]. A minimum of 50,000 cells were acquired in all sorting runs. The sorted transconjugant and recipient cells were lysed and DNA extractions were performed using the DNeasy Powersoil Pro Kit (QIAGEN®, Germany). 16S rRNA gene amplicon sequencing was conducted at the Australian Centre for Ecogenomics (ACE, Brisbane, Australia). In brief, the V3-V4 regions of the 16S rRNA were amplified using primer set 341F (5′-CCTAYGGGRBGCASCAG-3′) and 806R (5′-GGACTACHVGGGTWTCTAAT-3′) [17]. PCR amplifications were performed in NEBNext® UltraTM II Q5® Mastermix (New England Biolabs) under standard PCR conditions. The resulting PCR amplicons were purified using Agencourt AMPure XP beads (Beckman Coulter), followed by indexing with unique 8 bp barcodes using the Illumina Nextera XT 384 sample Index Kit A-D (Illumina FC-131–1002). Indexed amplicons were pooled together in equimolar concentrations and sequenced on a MiSeq (Illumina) in paired-end mode with V3 300 bp chemistry according to the manufacturer’s instructions.

### Analysis of sequencing data

Raw amplicon sequencing reads were analyzed using the DADA2 pipeline to obtain amplicon sequence variants (ASVs) [36]. In brief, the reads were filtered, trimmed, dereplicated, and merged. After discriminating biological sequences from sequencing errors, ASVs were inferred. The chimera-free reads were taxonomically classified using the SILVA small subunit (SSU) database (release 138) [37]. After sequencing, the total reads number was higher than 10,000 in gemfibrozil-, ibuprofen-, naproxen-, and propranolol-dosed transconjugant pools. Unfortunately, the total reads number was lower than 10,000 in carbamazepine- and iopromide-dosed transconjugant pools even after a few trials. Thus, the sequencing results under the exposure of carbamazepine or iopromide were not included in the study. All sequences obtained in this study were deposited in NCBI under accession number PRJNA746678.

### Analysis of genus-level permissiveness

Genus-level permissiveness was determined to quantitatively analyze bacterial uptake ability of the broad-host-range conjugative RP4 plasmid. It was calculated as described previously [38]. In brief, the relative abundance of each genus in the transconjugant pool was divided by that in the corresponding recipient microbial community. The resulting calculated permissiveness in the pharmaceutical-dosed groups was compared with that in the non-pharmaceutical control group. Fold changes of higher than 1 indicated non-antibiotic pharmaceuticals could enhance the permissiveness of a specific genus, and vice versa.

### Correlation tests

Correlation tests were conducted to identify whether the ROS production of donor or recipient bacteria was correlated with the conjugative transfer ratio. Pearson correlation was applied to calculate correlation coefficient *r*, followed by consulting the correlation coefficient table. The correlation was significant if *P* value was less than 0.05.

### Statistical analysis

Data were expressed as mean ± standard deviation. SPSS for Mac version 25.0 was applied for data analysis. Independent-sample *t* tests were performed, and the Benjamini–Hochberg correction method was applied for multiple comparisons [39]. *P* values less than 0.05 were considered to be statistically significant.

## Results

### Non-antibiotic pharmaceuticals can promote bacterial conjugation

To test whether non-antibiotic pharmaceuticals affect the horizontal transfer of IncP1-α plasmid-borne antibiotic resistance at a community-wide level, we characterized transfer dynamics from a *P. putida* donor to an activated sludge recipient community using fluorescence-based mating assays. The six non-antibiotic pharmaceuticals, including carbamazepine, gemfibrozil, ibuprofen, iopromide, naproxen, and propranolol were individually dosed at final concentrations of 0.5 mg/L (environmentally relevant [32, 33]), 5 mg/L, or 50 mg/L in each broth mating system to investigate whether these pharmaceuticals can affect conjugative transfer.

**Fig. 2:**
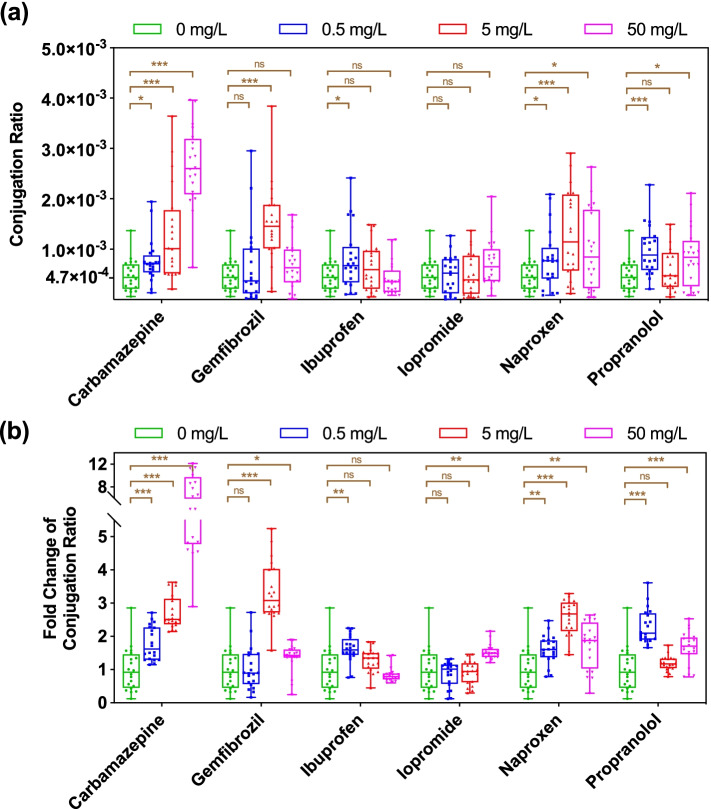
Conjugative transfer of plasmid-borne antibiotic resistance in activated sludge bacteria under exposure to six commonly consumed non-antibiotic pharmaceuticals. **a** Conjugation ratio. **b** Fold change of conjugation ratio compared with the solvent control. Significant differences between non-antibiotic pharmaceutical-dosed samples and the control were analyzed by independent-sample *t* test with Benjamini–Hochberg multiple comparison testing, * *P* < 0.05, ** *P* < 0.01, *** *P* < 0.001

Conjugative transfer of RP4 plasmid occurred in the 24 h mating assay in the absence of pharmaceuticals with transfer ratios in the range of 4.7 × 10^−4^ ± 3.0 × 10^−4^ transconjugants per recipient (Fig. 2a). The addition of carbamazepine and naproxen across all tested concentrations (0.5, 5, 50 mg/L), gemfibrozil at 5 and 50 mg/L, ibuprofen at 0.5 mg/L, and propranolol at 0.5 and 50 mg/L increased the conjugation ratio significantly (*P* < 0.05) (Fig. 2b). In detail, for carbamazepine, a dosage dependent increase in conjugation ratio was observed with the elevated levels of carbamazepine, and reached up to 7.1-fold with a maximum of 2.7 × 10^−3^ ± 7.7 × 10^−4^ transconjugant per recipient at 50 mg/L. In contrast, no dosage dependence was seen in conjugation ratio for other pharmaceutical-dosed groups. For example, in the naproxen-dosed group, conjugation ratio increased from 0 to 5 mg/L of naproxen, but decreased at the highest concentration (50 mg/L). For gemfibrozil, ibuprofen and propranolol plasmid transfer was significantly increased (*P* = 0.000007–0.049) for at least one, but not all concentrations. In contrast, iopromide did not cause any significantly enhanced conjugation ratios (*P* = 0.061–0.91). We also confirmed that the treatment with antibiotic chloramphenicol (Chl) significantly (*P* < 0.05) increased conjugation ratio at municipal sewage-relevant concentrations, with up to 3.5-fold induced at 0.1 mg/L Chl (Figure S3).

Collectively, the tested non-antibiotic pharmaceuticals, including the anticonvulsant carbamazepine, the anti-inflammatory drugs ibuprofen, naproxen, the lipid-lowering drug gemfibrozil, and the *β*-blocker propranolol, promoted the horizontal transfer of plasmid-borne antibiotic resistance in a complex community setting, with the X-ray contrast agent iopromide the lone exception. Notably, carbamazepine, ibuprofen, naproxen, and propranolol imposed significant impacts on plasmid transfer (*P* = 0.000000024–0.049) even at low, environmentally relevant concentrations (0.5 mg/L).

### Bacterial ROS production is associated with increased conjugation

**Fig. 3:**
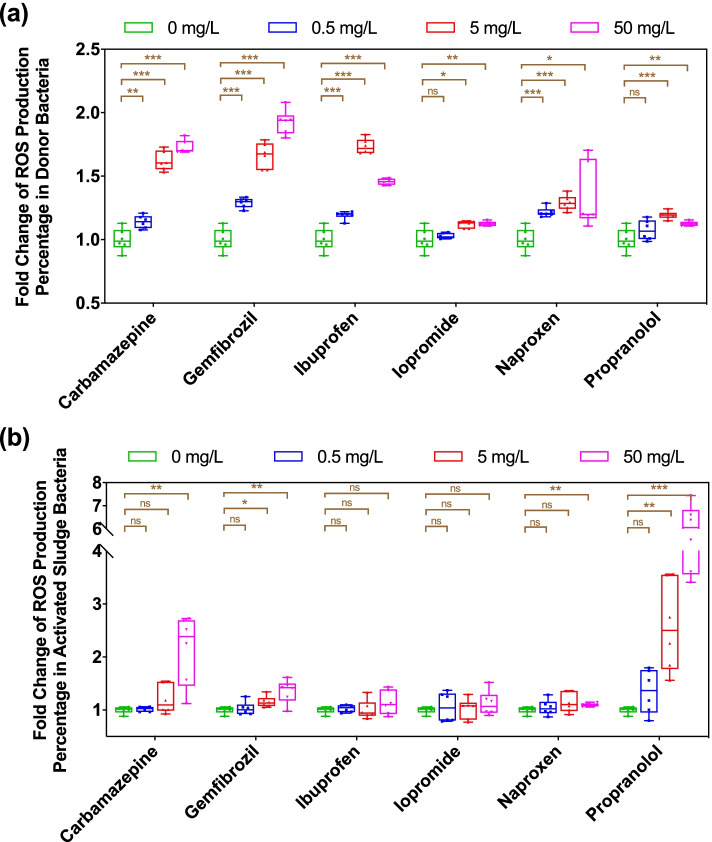
Effects of non-antibiotic pharmaceuticals on ROS production in donor and recipient bacteria. **a** Fold change of ROS production percentage in donor bacteria. **b** Fold change of ROS production percentage in recipient activated sludge bacteria. Significant differences between non-antibiotic pharmaceutical-dosed samples and the solvent control were analyzed by independent-sample *t* test with Benjamini–Hochberg multiple comparison testing, *P < 0.05, **P < 0.01, *** *P* < 0.001

ROS are natural byproducts of metabolism in bacteria, and their production may increase dramatically under some environmental stress, which might promote the horizontal transfer in several pure-culture model bacterial species [15, 16, 40]. To test the effects of non-antibiotic pharmaceuticals on ROS production at the microbial community level, we applied a fluorescence-based method to detect ROS levels. For the donor bacterium *P. putida* with RP4 plasmid, the ROS levels increased significantly under the exposure of 5 mg/L or 50 mg/L of all six non-antibiotic pharmaceuticals selected. The fold change ranged from 1.2- to 3.1-fold (*P* = 0.000000003–0.012) (Fig. 3a). The lowest concentration (0.5 mg/L) of all drugs showed less ROS production, with only a 1.1- to 1.3-fold increase (*P* = 0.00002–0.45). Regarding the activated sludge recipient bacteria, the total ROS production showed different trends. Specifically, carbamazepine, gemfibrozil, naproxen and propranolol at higher concentrations (i.e., 5 mg/L or 50 mg/L) increased the total ROS production significantly, and the fold change was as high as 5.6-fold (*P* = 0.00005, Fig. 3b). However, ibuprofen and iopromide did not affect the production of ROS in activated sludge significantly (*P* = 0.18–0.93, Fig. 3b). We also found the increased ROS generation in the mixture of donor and recipients (activated sludge) in the presence of the tested molecules (Text S3; Figure S4).

In addition, we conducted correlation tests between conjugative transfer ratio and ROS production in donor/recipient bacteria. We found that the total ROS production in recipient activated sludge bacteria is associated with conjugation. For example, high concentrations of ibuprofen or iopromide increased ROS production in donor bacteria significantly, but no significant enhancement was seen regarding the conjugation ratio, as they imposed negligible effects on total ROS production in recipient bacteria. Also, the correlations between conjugation ratio and ROS production in recipient activated sludge bacteria in other four groups were significant (*P* < 0.05).

### Non-antibiotic pharmaceuticals affect the transconjugant microbial community

To investigate the effects of non-antibiotic pharmaceuticals on the diversity of the microbial community in the recipient and the transconjugant pool, FACS isolation of transconjugant and recipient community, and subsequent 16S rRNA gene amplicon sequencing were applied.

**Fig. 4:**
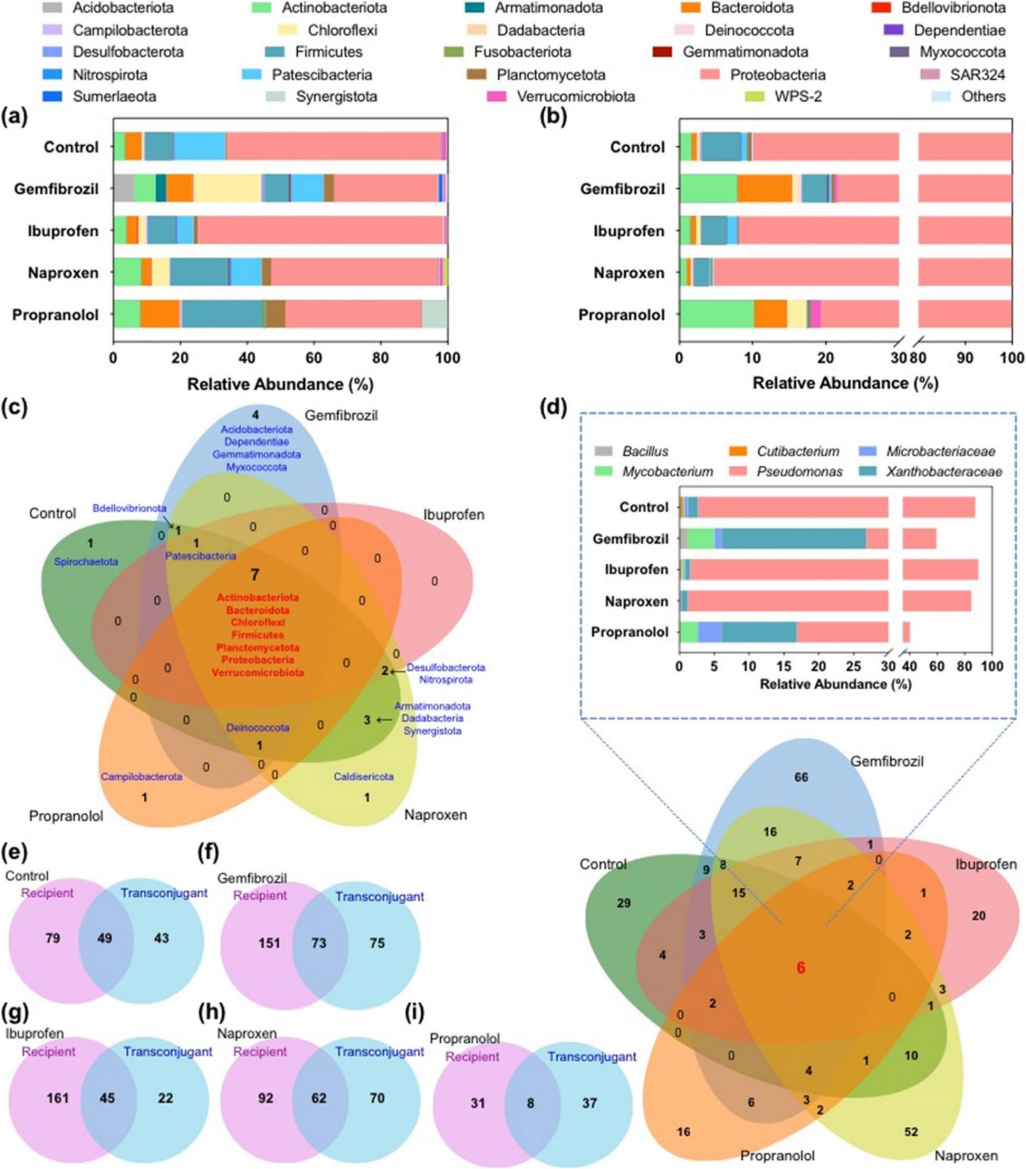
Microbial community composition in recipient and transconjugant pools. **a** Recipient microbial community composition at phylum level. **b** Transconjugant microbial community composition at phylum level. **c** Venn diagram of transconjugant pools in different non-antibiotic pharmaceutical-dosed groups at phylum level. The Venn diagram is presented as the number of phyla identified and the name of specific phylum. **d** Venn diagram of transconjugant pools in different non-antibiotic pharmaceutical-dosed groups at genus level. The Venn diagram is presented as the number of genera identified. The bar chart indicates relative abundance of the 6 kinds of shared genera in each transconjugant pool. **e**–**i** Venn diagram of the number of genera identified in recipient and the corresponding transconjugant pool in the non-pharmaceutical control, gemfibrozil, ibuprofen, naproxen, and propranolol group, respectively. The concentration for each non-antibiotic pharmaceutical was 50 mg/L

In the recipient microbial community, 38 phyla were identified (Fig. 4a). The relative abundance of each phylum varied in different pharmaceutical-dosed groups, ranging from < 0.1% to > 50%. In the transconjugant pools, as expected, Proteobacteria showed the highest relative abundance in all transconjugant pools, which was in line with the predicted IncP1 plasmid host range [41]. This may be because the donor bacterium also belongs to the Proteobacteria. In addition to Proteobacteria, another 22 further phyla were identified as transconjugants able to take up the conjugative plasmid RP4, including Actinobacteria, Firmicutes and Verrucomicrobiota. However, the transconjugant phyla identified across the different pharmaceutical-dosed groups varied, with only 7 common core phyla: Actinobacteriota, Bacteroidota, Chloroflexi, Firmicutes, Planctomycetota, Proteobacteria, and Verrucomicrobiota (Fig. 4c). Apart from these 7 core phyla, only 2 and 3 additional phyla were detected in propranolol- and ibuprofen-treated transconjugant pools, while as many as 9 additional phyla were detected in naproxen-dosed transconjugant pools. Some phyla were only identified in one of the transconjugant pools; for instance Caldisericota was only identified in the naproxen-dosed transconjugant pool, while Campilobacterota was only identified in the propranolol-dosed transconjugant pool.

Notably, the microbial composition in each transconjugant pool differed from their corresponding recipient community (Fig. 4a, b). For example, the relative abundance of the most abundant phylum (Proteobacteria) increased in transconjugant pools, even in the control treatment. In the non-pharmaceutical control group, the relative abundance of Proteobacteria was 64% in the recipient microbial community, and this increased to 89% in the corresponding transconjugant pool. Similar trends were also seen in the pharmaceutical-dosed groups, with an increase from 31 to 78%, 72 to 92%, 49 to 95%, and 40 to 80% in gemfibrozil-, ibuprofen-, naproxen-, and propranolol-dosed groups, respectively. In addition, when exposed to different non-antibiotic pharmaceuticals, some other phyla in transconjugant pools exhibited various changes compared with the corresponding recipient microbial community. For example, in the gemfibrozil-dosed group, the relative abundance of Chloroflexi decreased from 20% in the recipient to 1% in the transconjugant pools. Interestingly, the bacterial composition in ibuprofen- and naproxen-dosed transconjugant pools was similar to that in the control transconjugant pool, with Proteobacteria occupying 90% of the total microbial community, which was higher than that of other pharmaceutical-dosed transconjugant pools.

It should be noted that the absolute number of donor bacteria was not significantly changed when challenged with these non-antibiotic pharmaceuticals [42]. This indicated bacterial community variation was due to the influence of external non-antibiotic pharmaceuticals, rather than bacterial number.

We further investigated the microbial composition in each recipient and transconjugant pool at the genus level. A total of 289 genera were identified in all transconjugant pools. Among all the five transconjugant pools (non-pharmaceutical control-, gemfibrozil-, ibuprofen-, naproxen-, and propranolol-dosed groups), 6 genera were identified in all pools. These 6 shared genera included pathogens and opportunistic pathogens such as *Bacillus*, *Cutibacterium*, *Mycobacterium*, *Pseudomonas* [43–45], and occupied more than 40% of relative abundance in each transconjugant pool (Fig. 4d). Genus *Pseudomonas* showed the highest abundance in all transconjugant pools. The relative abundance of *Pseudomonas* was 85%, 33%, 88%, 84%, and 23% in the non-pharmaceutical control, gemfibrozil-, ibuprofen-, naproxen-, and propranolol-dosed groups, respectively. Interestingly, we found that gemfibrozil- and propranolol-dosed groups showed lower *Pseudomonas* abundance and higher abundance of the other shared genera, which may contribute to their higher absolute observed conjugative transfer ratio.

Regarding the total number of genera found in each transconjugant pool, the gemfibrozil-dosed group showed the highest number. Some genera were only identified in the recipient microbial community, while others were only identified in the transconjugant pool (Fig. 4e–i). The genera only appearing in the recipient pool were the bacteria that did have received the conjugative plasmid from the donor, but probably at a lower ratio. Those genera only appearing in transconjugant pools may be associated with their relative abundance in the recipient community being too low to be identified. Consequently, an increased relative abundance and detection in transconjugant pools indicated these genera were highly permissive towards the antibiotic resistant plasmid.

Notably, several microbes with high pathogenic potential, including *Acinetobacter* and *Roseomonas*, were not identified in the transconjugant pool of the non-pharmaceutical control, but were identified in all pharmaceutical-dosed groups. This indicated that non-antibiotic pharmaceuticals could specifically elevate the plasmid uptake ability of such pathogenic microbes. Moreover, in addition to the above-mentioned relative abundance of transconjugant or recipient bacterial community under the exposure of non-antibiotic pharmaceuticals, the absolute number of donor bacteria was not significantly changed when challenged with these non-antibiotic pharmaceuticals [46]. This indicated bacterial community variation was due to the influence of external non-antibiotic pharmaceuticals, rather than bacterial number.

### Non-antibiotic pharmaceuticals modulate community plasmid uptake ability

**Fig. 5:**
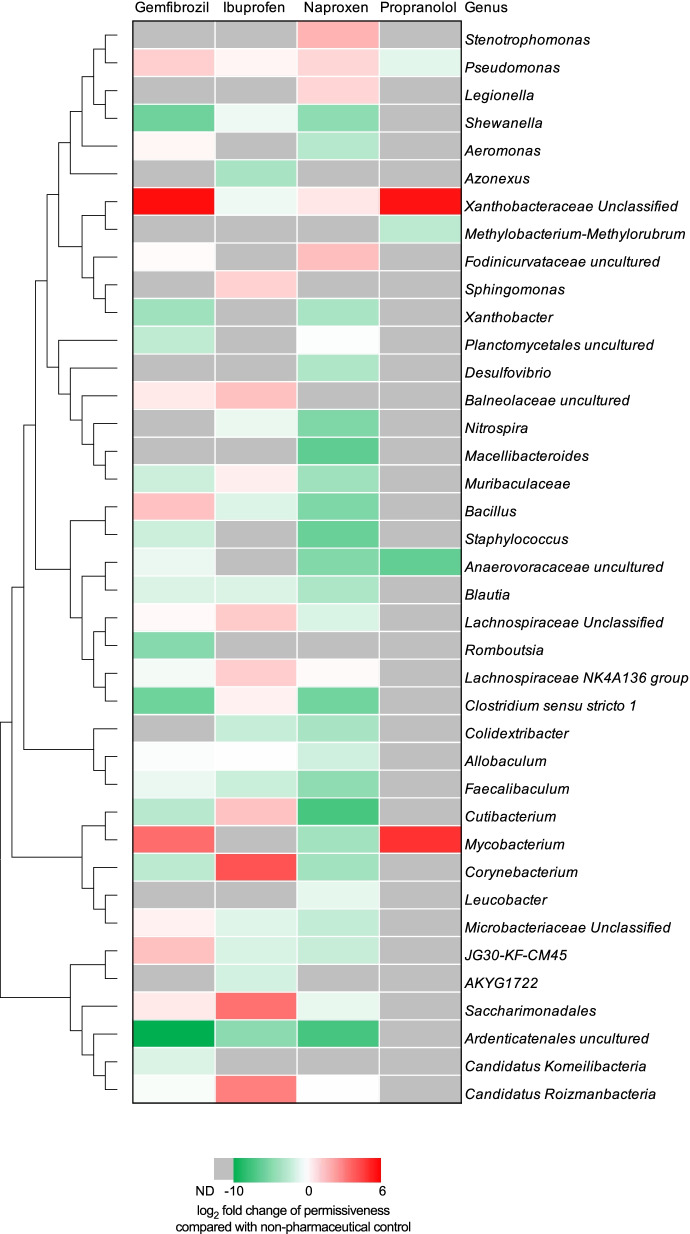
Tree clustering for 39 genera with calculated permissiveness. Heatmap is shown as the log_2_ fold change of permissiveness in non-antibiotic pharmaceutical-dosed groups, compared with the non-pharmaceutical control. Permissiveness can only be calculated when the genus was identified in both transconjugant pool and the corresponding recipient community. Genera with calculated permissiveness in the non-pharmaceutical control and in at least one pharmaceutical-dosed group are shown. ND indicates the genus which was only identified in either recipient community or transconjugant pool. The concentration for each non-antibiotic pharmaceutical was 50 mg/L

In order to quantitively investigate the changes in relative abundance of each genus under the exposure of non-antibiotic pharmaceuticals, we analyzed the microbial uptake ability of the broad-host-range conjugative RP4 plasmid in non-antibiotic pharmaceutical-dosed groups, and compared it with the non-pharmaceutical control group. To this end, genus-level permissiveness was compared [38]. Permissiveness was calculated as the genus relative abundance in transconjugant pool divided by that in the corresponding recipient community. Only the permissiveness of genera that were identified in both the transconjugant pool and recipient community simultaneously could be determined. After calculating and comparing the permissiveness in pharmaceutical-dosed groups and the non-pharmaceutical control group, 39 genera were screened (with calculated permissiveness in the non-pharmaceutical control and in at least one pharmaceutical-dosed group shown). We found that only the permissiveness of *Xanthobacteraceae* showed an identical trend in all pharmaceutical-dosed groups, with elevated permissiveness up to a 53.4-fold increase compared with the control group (Fig. 5). In all drug treatments except propranolol, the permissiveness of *Ardenticatenales* decreased up to 1659.7-fold. On the contrary, fold change of other genera’s permissiveness showed various trends in different non-antibiotic pharmaceutical-dosed groups. For example, the permissiveness of *Mycobacterium* increased in gemfibrozil- and propranolol-dosed groups, 11.2- and 28.8-fold, respectively; while it decreased in the naproxen-dosed group. Also, the permissiveness of *Bacillus* only increased in the gemfibrozil-dosed group, while it decreased or was not detected in the other three groups. Interestingly, we found that regarding the number of genera with enhanced permissiveness compared to the non-pharmaceutical control, the gemfibrozil-dosed group showed the most, with 11 genera. While in the naproxen-dosed group there were only 6 genera with enhanced permissiveness. The lower permissiveness may also explain why 50 mg/L naproxen showed lower phenotypic conjugative transfer ratio compared with gemfibrozil. Collectively, quantitative permissiveness analyses indicated non-antibiotic pharmaceuticals could modulate the community uptake ability towards the RP4 plasmid at genus level, and the modulation effect varied when exposed to different non-antibiotic pharmaceuticals.

## Discussion

Antibiotic resistance is a major threat to both public and environmental health. HGT is one of the critical pathways for dissemination of antibiotic resistance in bacterial communities [1, 47]. As broad-host-range conjugative plasmids are usually isolated from soil environments and can be detected in various environments [48–52], plasmid-mediated conjugation is shown to happen in natural environments spontaneously, with a ratio of about 1 in every 10^6^–10^7^ cells [6]. It is thus one of the major pathways for the spread and evolution of antibiotic resistance in the environment [1, 47]. Antibiotics are shown to promote this process [53, 54], while the contributions by non-antibiotic pharmaceuticals, which occupy 95% of the drug market [11], are largely overlooked. Recently, we demonstrated that several commonly consumed non-antibiotic pharmaceuticals can facilitate antibiotic resistance dissemination via both conjugation and transformation in pure-culture bacterial models [15, 16]. However, their effects on mixed-culture environments remain unknown. In this study, we demonstrated that non-antibiotic pharmaceuticals can promote the spread of antibiotic resistance in mixed-culture environmental microbial communities.

We employed activated sludge from wastewater treatment plants as the environmental sample, as it is the reservoir for a variety of bacteria and environmental pollutants include non-antibiotic pharmaceuticals [18, 19, 32]. By applying a fluorescently *mCherry*-tagged environmental bacterium, *P. putida*, with a broad-host-range self-transmissible IncP-*α* RP4 plasmid (resistant to ampicillin, kanamycin, and tetracycline), we could visualize and quantify conjugative transfer in activated sludge samples. Notably, the donor bacterium (*Pseudomonas*) was not exotic to the recipient community as it was detected in the activated sludge samples. This, together with the culture medium comprising sterile bioreactor feed, means this conjugation system is likely to reflect processes that occur in the environment such as wastewater treatment plants. The phenotypic microscopy detection indicated that commonly consumed and detected non-antibiotic pharmaceuticals, including carbamazepine, gemfibrozil, ibuprofen, naproxen, and propranolol, at environmentally relevant concentrations [22, 23], could increase the conjugative transfer of plasmid-borne ARGs. The fold change was up to 7.1-, compared with the non-pharmaceutical control. Similar phenomena were previously elucidated in pure-culture studies, where except for iopromide, the remaining five non-antibiotic pharmaceuticals increased the horizontal transfer of antibiotic resistance genes via conjugation or transformation [15, 16]. However, the observed effect concentrations of the pharmaceuticals that promoted gene transfer were different when comparing single species experiments and mixed-culture results observed here. This is best explained by the fact that in a complex microbial community, various recipient bacterial species may respond differently to these non-antibiotic pharmaceuticals, compared to pure culture conditions where all bacterial cells of one species react similarly.

The non-antibiotic pharmaceuticals also induced elevated ROS production in donor and recipient activated sludge bacteria. Compared with the other five non-antibiotic pharmaceuticals, iopromide caused the least enhanced ROS production in the donor strain, which was consistent with our previous study [16]. Considering ROS have been demonstrated to play a key role during the enhanced conjugation in pure-culture bacterial models [15, 34, 40, 55], we inferred that the overproduction of ROS caused by these non-antibiotic pharmaceuticals contributed to enhanced conjugation in mixed-culture activated sludge samples. Our previous studies employed a series of methods, including oxidative stress and cell membrane permeability quantifications DNA sequencing, genome-wide RNA sequencing, and proteomic analysis. We found that ROS and upregulation of conjugative transfer relevant genes located on plasmid are the major contributors to pharmaceutical-enhanced conjugative transfer [15, 42].

We then investigated recipient and transconjugant communities under the exposure of non-antibiotic pharmaceuticals, by applying FACS and 16S rRNA gene amplicon sequencing. Sequencing results indicated that the green-fluorescent transconjugant pools differed from their corresponding recipient communities (Fig. 4b). Within-genus conjugation was dominant in all groups (both the control and non-antibiotic pharmaceutical-dosed groups), yet conjugation also occurred across genera and even across phyla. This was evidenced by the fact that Proteobacteria and *Pseudomonas* (what the donor bacterium belongs to) had the highest relative abundance at phylum and genus level, respectively. This is likely to be because the donor bacterium was a strain of *Pseudomonas*, thus it was easier for the conjugative plasmid transfer within its own bacterial genus, due to a shorter phylogenetic distance [56]. In addition, transconjugants extended to another 22 phyla, with a total of 289 genera identified in all transconjugant pools, including pathogens and opportunistic pathogens such as *Acinetobacter*, *Clostridium*, *Corynebacterium*, *Escherichia-Shigella*, *Mycobacterium*, and *Legionella* [43–45]. This further demonstrated that broad-host-range plasmid-borne ARGs can spread widely among environmental microbial communities [3, 20, 57]. Moreover, we found that non-antibiotic pharmaceuticals could modulate the transconjugant microbial community, when comparing the transconjugant pools under different conditions. Among all the genera identified, 6 types of genera were found in all transconjugant pools, while other genera were different in various non-antibiotic pharmaceutical-dosed groups. This may be due to different mechanisms of action of these non-antibiotic pharmaceuticals [58], thus imposing various metabolic fitness in microbial communities [59]. The modulated transconjugant microbial communities may also contribute to the enhanced phenotypic conjugative transfer.

We further quantitatively analyzed the community uptake ability (i.e., permissiveness) towards this broad-host-range IncP-*α* RP4 plasmid. Exposure to non-antibiotic pharmaceuticals could increase or decrease the genus-level permissiveness depending on the type of pharmaceutical and genus. This was shown from different genus-level permissiveness when compared with the non-pharmaceutical control. Also, variations were seen in different non-antibiotic pharmaceutical-dosed groups. Permissiveness analyses demonstrated non-antibiotic pharmaceuticals could affect microbial uptake ability towards the conjugative plasmid, enabling some genera (*Acinetobacter*, *Mycobacterium*, and *Xanthobacteraceae*) to become antibiotic resistant more easily, while they could suppress some genera (*Ardenticatenales*, *Faecalibaculum* and *Shewanella*) to uptake plasmid-borne ARGs. Thus, microbial competitiveness during the evolution of antibiotic resistance was also modulated under the exposure of non-antibiotic pharmaceuticals. Notably, *Aeromonas* is one of the specific genera that plays a significant role in facilitating transfer and maintenance of plasmid-mediated ARGs in various environmental communities [17, 60, 61]. In this study, *Aeromonas* was also identified in the transconjugant pools from the control, gemfibrozil-, and naproxen-dosed groups, and gemfibrozil could enhance its permissiveness. On the contrary, as *Aeromonas* was not detected in ibuprofen- or propranolol-dosed group, these non-antibiotic pharmaceuticals reduced permissiveness. In addition to the non-antibiotic pharmaceuticals used in this study, community permissiveness has also been reported to be sensitive to environmental metal(loid) stress, with increased or decreased permissiveness with different metals and doses [38]. However, such high permissiveness among the genera may not be always the case under different conditions. The detailed relationships between genus-level permissiveness and specific environmental contaminants need further investigations. Moreover, pharmaceuticals such as carbamazepine can show antimicrobial activity on different bacterial species [62, 63]. Increased plasmid permissiveness could be also due to the pharmaceutical-mediated selection of highly permissive recipient cells among the microbial community. Further studies about whether and how the tested pharmaceuticals favor the highly permissive species are expected to comprehensively clarify their roles in plasmid-mediated conjugative transfer among the microbial community.

## Conclusion

We reported that several commonly consumed and detected non-antibiotic pharmaceuticals, including an anticonvulsant, nonsteroidal anti-inflammatory drugs, a lipid-lowering drug, and a *β*-blocker, could facilitate the spread of plasmid-borne antibiotic resistance in a model system similar to wastewaters. The non-antibiotic pharmaceuticals not only change the transconjugant microbial community, but also modulate the community uptake ability towards the broad-host-range conjugative plasmid at the genus level. The enhanced ROS generation caused by non-antibiotic pharmaceuticals may play an important role in the promoted transfer of ARGs. This study warns of the contribution of non-antibiotic pharmaceuticals to dissemination and evolution of antibiotic resistance in the environment at the community level. Note that the fluorescence-based techniques used in this study have weaknesses such as failure to detect or sort bacterial species that exhibit a low activity of the promoter for *gfp* expression [64]. More additional advanced techniques will be needed to sort out poorly/non-fluorescent transconjugants. In addition, further studies are needed to investigate the effects of non-antibiotic pharmaceuticals on modulating the evolutionary host range for conjugative plasmids and specifically whether and how these molecules select permissive species within the microbial communities.

## Supplementary Information

**Additional file 1**: **Text S1.** Stability of RP4 plasmid. **Text S2.** Effect of antibiotic chloramphenicol (Chl) on conjugative transfer. **Text S3.** ROS production in the mixed culture. **Figure S1.** Plasmid stability in bacterial cells. a, Set-up of plasmid stability assays over 5 days. In each cycle, 1% of cultures were grown in fresh LB medium. b, Dynamic analysis of plasmid stability in LB media. **Figure S2.** FACS sorting of transconjugant cells from a mixture initiated with activated sludge and *P. putida* KT2440 carrying *gfp*-RP4 plasmid. Gate I sorts for bacterial size based on forward-H and side scatter-H; Gate II sorts for singlet based on forward-H and forward-W; and Gate III selects only those green cells (left corner) [1]. Panel (a) shows the results from the donor, panel (b) shows the results from positive control (green transconjugants), while panel (c) shows the results from activated sludge. **Figure S3.** Fold change in conjugative transfer of plasmid-borne antibiotic resistance in activated sludge bacteria under exposure to antibiotic Chl (*N* = 6). Significant differences between carbamazepine-treated groups and the control groups were analyzed by Independent-sample *t *test and the Bonferroni correction, * *P* < 0.05 and ** *P *< 0.01. **Figure S4.** Fold changes in ROS production in the mixed culture (the donor and activated sludge) under exposure to carbamazepine. Significant differences between the control and the treated groups were tested with Independent-sample *t* test and the Bonferroni correction, * *P* < 0.05 and ** *P* < 0.01.

## Data Availability

The datasets generated during and/or analyzed during the current study are available in NCBI under accession number PRJNA746678.

## Abbreviations

ARGs: Antibiotic resistance genes
CLSM: Confocal laser scanning microscope
DCFDA: 2′,7′-Dichlorofluorescein diacetate
DMSO: Dimethyl sulfoxide
FACS: Fluorescence-activated cell sorting
HGT: Horizontal gene transfer
ROS: Reactive oxygen species
SBR: Sequencing batch reactor
SSU: SILVA small subunit
TCOD: Total chemical oxygen demand
