# Supplementary material for: Non-antibiotic pharmaceuticals promote conjugative plasmid transfer at a community-wide level
Source: Microbiome. 2022 Aug 12;10:124. doi: 10.1186/s40168-022-01314-y (PMC9373378; doi:10.1186/s40168-022-01314-y)
Supplement: Supplementary file 2 — Additional file 1: Text S1. Stability of RP4 plasmid. Text S2. Effect of antibiotic chloramphenicol (Chl) on conjugative transfer. Text S3. ROS production in the mixed culture. Figure S1. Plasmid stability in bacterial cells. a, Set-up of plasmid stability assays over 5 days. In each cycle, 1% of cultures were grown in fresh LB medium. b, Dynamic analysis of plasmid stability in LB media. Figure S2. FACS sorting of transconjugant cells from a mixture initiated with activated sludge and P. putida KT2440 carrying gfp-RP4 plasmid. Gate I sorts for bacterial size based on forward-H and side scatter-H; Gate II sorts for singlet based on forward-H and forward-W; and Gate III selects only those green cells (left corner) [1]. Panel (a) shows the results from the donor, panel (b) shows the results from positive control (green transconjugants), while panel (c) shows the results from activated sludge. Figure S3. Fold change in conjugative transfer of plasmid-borne antibiotic resistance in activated sludge bacteria under exposure to antibiotic Chl (N = 6). Significant differences between carbamazepine-treated groups and the control groups were analyzed by Independent-sample t test and the Bonferroni correction, * P < 0.05 and ** P < 0.01. Figure S4. Fold changes in ROS production in the mixed culture (the donor and activated sludge) under exposure to carbamazepine. Significant differences between the control and the treated groups were tested with Independent-sample t test and the Bonferroni correction, * P < 0.05 and ** P < 0.01. [file 40168_2022_1314_MOESM1_ESM.docx]

Supplementary Information

**Non-antibiotic pharmaceuticals promote conjugative plasmid transfer at a community-wide level**

Yue Wang^a,1^, Zhigang Yu^a,1^, Pengbo Ding^a^, Ji Lu^a^, Uli Klümper^b^, Aimee K. Murray^c^, William H. Gaze^c^, Jianhua Guo^a,^*.

^a^ Australian Centre for Water and Environmental Biotechnology (ACWEB, formerly AWMC), The University of Queensland, Brisbane, Queensland 4072, Australia

^b^ Institute for Hydrobiology, Technische Universität Dresden, Dresden 01217, Germany

^c^ European Centre for Environment and Human Health, University of Exeter Medical School, Environment & Sustainability Institute, Penryn Campus, TR10 9FE, United Kingdom

* Corresponding author: [jianhua.guo@uq.edu.au](mailto:jianhua.guo@uq.edu.au)

^1^ These authors contributed equally to the work.

This file includes

Texts 1 to 3

Figures 1 to 4

References

**Text S1. Stability of RP4 plasmid**

To examine whether the plasmid could be lost from the donor, plasmid stability assay for RP4 in *P. alloputida* was conducted in LB media [1] containing 0.5 mg/L carbamazepine (as a representative) (Fig. S1a). A negative control was prepared following the same procedure but added aliquots of DSMO solvent. A positive control was also prepared by adding 0.5 mg/L Tet (little killing effect). The total cell number was enumerated from the plate that did not contain Tet, while the plasmid-bearing cell number was counted from the plate that contained 10 mg/L Tet. Each sample was prepared in biological triplicate and the plates were prepared in biological triplicate and technical duplicate.

**Text S2. Effect of antibiotic chloramphenicol (Chl) on conjugative transfer**

The mix culture was exposed to antibiotic Chl with different concentrations (0, 0.01, 0.05, and 0.1 mg/L), which covered the range of Chl concentration in municipal sewage [2]. After 24 h mating at room temperature, the samples were suspended by a vortex, centrifuged at 400 × g for 5 min to remove large residues, and was then diluted 10 times by PBS before analysis. Conjugation events were quantified by a CytoFLEX S flow cytometer (Beckman Colter, USA) with excitation at 488 nm and emission at 525 nm (*gfpmut3b*) [3]. The results were dealt with FlowJo 7.6. Transconjugants were sorted based on triple gates (Fig. S2): the gate of forward scatter-H vs side scatter-H plot was initially set up to focus the particles with bacteria size; the gate of forward scatter-H vs forward scatter-W plot was used to target on singlet; the third gate of 561_TexaRed-A vs 488_SYBR-A plot was used to exclude any auto-fluorescent particles from activated sludge and at the same time sort only out transconjugants. Method of triple-gated sorting was confirmed by both the donor and the isolated transconjugant (from conjugation between pure culture) that only carried *gfp*-tagged RP4 plasmid, as well as activated sludge bacteria. The recipient number was also quantified as the event number detected on the down left quadrant. The conjugative transfer ratio was calculated as the transconjugant number divided by the total recipient number. Over 300,000 events in total were detected and were analyzed for all the samples. Each sample was prepared at least in six biological replicates.

**Text S3. ROS production in the mix culture**

The donor strain *P. alloputida* that carried *gfp*-tagged conjugative plasmid and chromosomal *mCherry* was overnight incubated at 30 ℃, in the presence of 10 mg/L tetracycline. Cell pellets were washed with PBS three times and was resuspended in the feeding medium of the bioreactor. The activated sludge was sonicated 30 min until mixed with the donor cells (1:1, v/v). Initially, the mixture was stained with DCFDA for 30 min at room temperature, in the darkness. The drug carbamazepine was then added to the mixed culture with different final concentrations (0.5, 5, and 50 mg/L). The control group was also prepared by adding solvent DMSO. After 2 h, the mixture was centrifuged at 400 × g for 5 min to remove large residues and were diluted 100 times with PBS solution before flow cytometry analysis of ROS production. Each sample was prepared in six biological replicates.


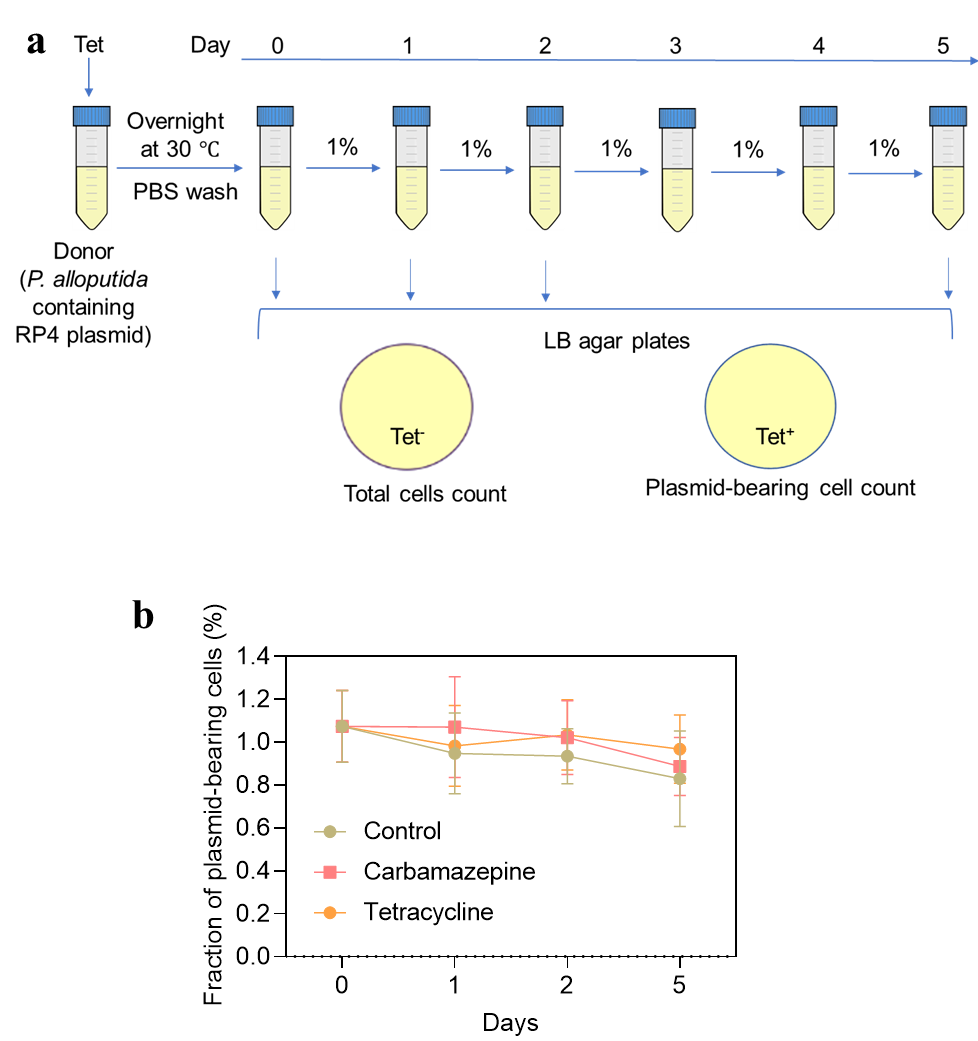


**Fig. S1.** Plasmid stability in bacterial cells. **a**, Set-up of plasmid stability assays over 5 days. In each cycle, 1% of cultures were grown in fresh LB medium. **b**, Dynamic analysis of plasmid stability in LB media.


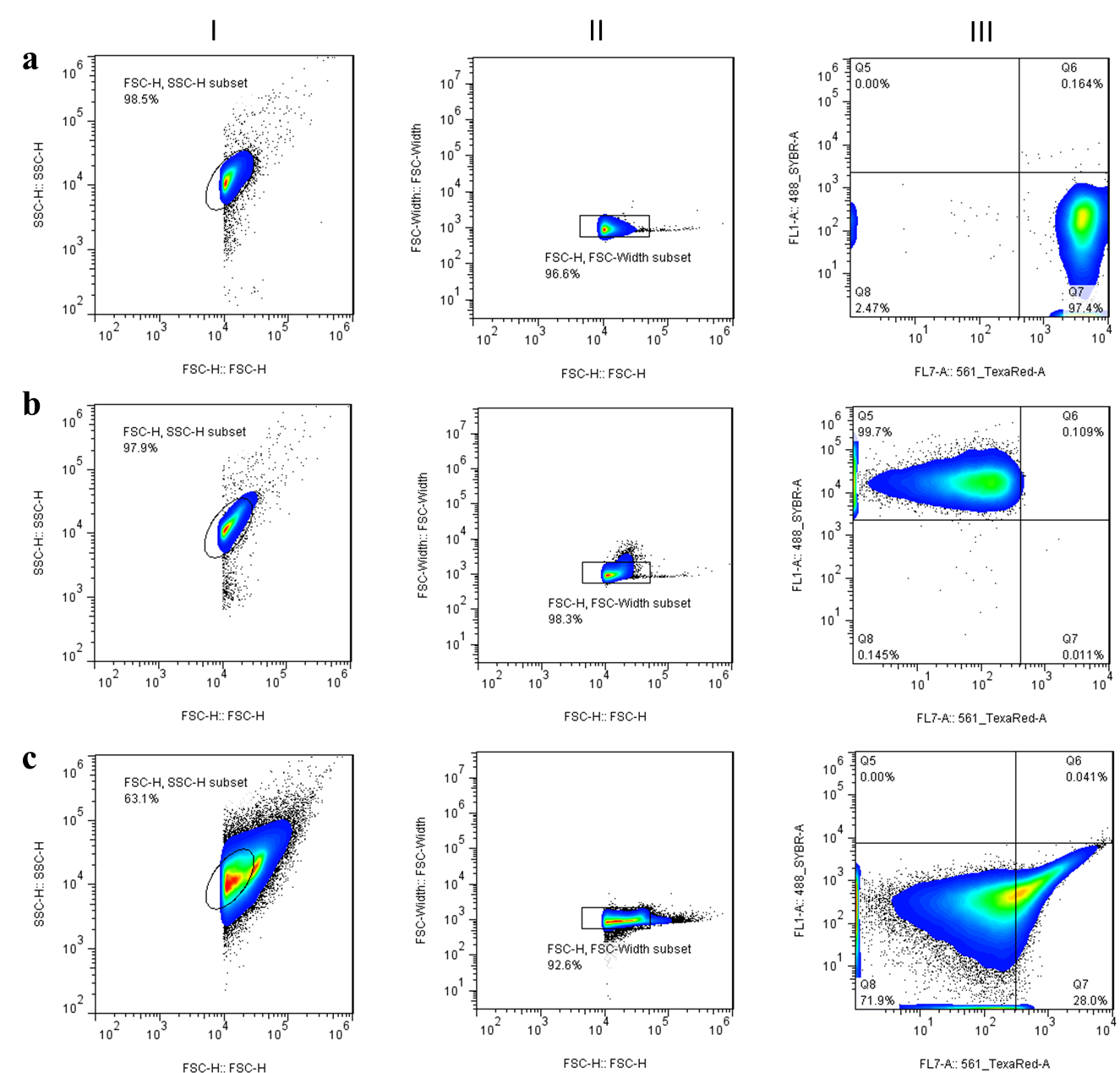


**Fig. S2.** FACS sorting of transconjugant cells from a mixture initiated with activated sludge and *P. putida* KT2440 carrying *gfp*-RP4 plasmid. Gate I sorts for bacterial size based on forward-H and side scatter-H; Gate II sorts for singlet based on forward-H and forward-W; and Gate III selects only those green cells (left corner) [1]. Panel (**a**) shows the results from the donor, panel (**b**) shows the results from positive control (green transconjugants), while panel (**c**) shows the results from activated sludge.

**Fig. S3.** Fold change in conjugative transfer of plasmid-borne antibiotic resistance in activated sludge bacteria under exposure to antibiotic Chl (*N* = 6). Significant differences between carbamazepine-treated groups and the control groups were analyzed by Independent-sample *t* test and the Bonferroni correction, * *P* < 0.05 and ** *P* < 0.01.

**Fig. S4.** Fold changes in ROS production in the mixed culture (the donor and activated sludge) under exposure to carbamazepine. Significant differences between the control and the treated groups were tested with Independent-sample *t* test and the Bonferroni correction, * *P* < 0.05 and ** *P* < 0.01.

**References**

1. Yu Z, Wang Y, Henderson IR, Guo J: Artificial sweeteners stimulate horizontal transfer of extracellular antibiotic resistance genes through natural transformation. *ISME J* 2022, 16:543-554.

2. Liu H, Zhang G, Liu C-Q, Li L, Xiang M: The occurrence of chloramphenicol and tetracyclines in municipal sewage and the Nanming River, Guiyang City, China. *J Environ Monitor* 2009, 11:1199-1205.

3. Klümper U, Riber L, Dechesne A, Sannazzarro A, Hansen LH, Sørensen SJ, Smets BF: Broad host range plasmids can invade an unexpectedly diverse fraction of a soil bacterial community. *ISME J* 2015, 9:934-945.
